# Profile of Male Breast Cancer in Makkah Region of Saudi Arabia: A 4-Year Retrospective Analysis of Radiology and Histopathology

**DOI:** 10.1155/2022/8831011

**Published:** 2022-06-22

**Authors:** Muhammad Saeed, Bothaina Mohammed Abdulshakour, Najwa Abdalkabeer A. Bantan, Afnan Hisham Falemban, Munir Abdulla, Ehab M. Melibary, Ahmad H. Mufti, Mohiuddin M. Taher

**Affiliations:** ^1^Department of Radiology, Al-Noor Specialty Hospital, Makkah, Saudi Arabia; ^2^Histopathology Division, Department of Laboratory Medicine, Al-Noor Specialty Hospital, Makkah, Saudi Arabia; ^3^Umm-Al-Qura University College of Medicine, Makkah, Saudi Arabia; ^4^Epidemiology and Infection Control Department, King Khaled University Medical Center, Abha, Saudi Arabia; ^5^Department of Medical Genetics, College of Medicine, Umm Al-Qura University, Makkah, Saudi Arabia; ^6^Science and Technology Unit and the Deanship of Scientific Research, Umm Al-Qura University, Makkah, Saudi Arabia

## Abstract

**Background:**

Mammography is a method widely used for the diagnosis of breast disorders in women and may help detect breast cancer in its early stages. Male breast cancer often remains undiagnosed or is poorly controlled until serious complications arise; therefore, the use of screening methods is needed to help with early diagnosis.

**Methods:**

From a total of 1,667 registered mammography cases screened, 17 male breast disease cases were included in this study. Mammography and ultrasound data were analyzed by Statistical Package of Social Sciences v.22 (SPSS). Diagnosis was made following biopsy in suspicious cases, and histopathological and immunological findings of all such patients were obtained for final diagnosis.

**Results:**

The mean age of the patients was 35 years (range, 14-70 years); 17.6% of the cases were aged 37 yrs, and 2 cases were aged 51 and 52 yrs. Of the 17 cases, 11 had breast lesions, and skin thickening was observed in only 1 case. The different patterns of lesions detected were asymmetry of the parenchyma, mastitis, and hamartoma (*n* = 1 each), malignant lesions (*n* = 2), and gynecomastia (*n* = 6). According to the BI-RADS categorization, 8 cases were benign, one case was probably benign, and 2 cases were likely malignant. In the 2 cases with malignant lesions, pathological diagnosis was made after hematoxylin and eosin and immunocytochemistry examination as invasive ductal carcinoma (IDC) of no special type (NST), grade II and grade III.

**Conclusions:**

Most breast lesions in this study population were benign, while IDC was the most common malignancy encountered. Mammography is currently the most accurate and cost-effective method for detecting breast lesions. The findings of our study may help increase awareness of male breast cancer and encourage Saudi men at risk to perform self-breast exam and undergo routine breast screening.

## 1. Introduction

Breast cancer is the most prevalent cancer among women in the Kingdom of Saudi Arabia (KSA), as well as other parts of the world [1]. Arabs share common demographic characteristics that include high levels of consanguinity, large family size and, recently, rapid population growth, particularly in KSA [2]. Worldwide, there were ~2.1 million newly diagnosed female breast cancer cases in 2018, accounting for almost 1 in 4 cancer cases among women (http://www.uicc.org/new-global-cancer-data-globocan-2018). Screening mammography is routinely offered to women for detection of breast cancer in several countries [3]. Over the last decade, several breast cancer epidemiological studies were conducted, awareness survey reports were published, and media events, such as the “Online Campaign To Raise Public Awareness About This Disease, Early Detection” were also organized in the Kingdom of Saudi Arabia [4–8]. According to a recently published news report, 2,240 women and 42 men were diagnosed with breast cancer in Saudi Arabia in 2019 [9]. Male breast cancer (MBC) is a rare malignancy, with an estimated incidence rate of ~1% of all breast cancer cases and <0.1% of all male cancers [10, 11]. According to the National Cancer Institute estimates, the lifetime risk of MBC is ~1 in 833, and ~2,620 men were reported to be diagnosed with breast cancer, and 520 men died from this disease in 2020 [12].

Breast cancer risk in men is increased by hereditary and genetic factors, including a personal or family history of breast or ovarian cancer [13, 14]. The risk of MBC increases 2.0-fold and 10.0-fold with one and two positive first-degree relatives, respectively [15]. One of the explanations for the cause of MBC is elevated sex hormone levels similar to those in women; some studies have found that AR positivity correlates with favorable outcomes [13, 16]. Incidences of MBC were reported in response to estrogen therapy in prostate cancer patients; however, having prostate cancer itself does not increase the chance of developing breast cancer [17]; it is even rare to have prostate and breast tumors present synchronously in a male patient [18]. Previous studies have suggested that an increased androgen receptor positivity is a risk factor in the development of breast cancers in female-male transexuals and few cases of breast cancer were reported in transgender males receiving testosterone therapy for gender-affirming hormonal treatment [19, 20]. Evidence exists to suggest that X chromosome can play a role in the neoplastic transformation of male breast epithelium, and X chromosome gain is paralleled by AR gene polysomy [21, 22]. The potential role of X chromosome gains in the neoplastic transformation of the male breast epithelium was also evidenced from the study of Klinefelter syndrome patients, where the male patient has an extra chromosome (47XXY) [23].

BRCA1 mutations seem to play a smaller role in MBC compared with female breast cancer [24], as BRCA2 mutations are more significantly associated with MBC than BRCA1 [25, 26]. MBC in patients with BRCA2 mutations tends to present at a younger age and may be associated with a poorer survival. Another study reporting the BRCA-status of MBC patients found that HER-2-positive MBC patients are known to carry BRCA2 mutations [27]. Patients with MBC are also more frequently hormone receptor- (HR-) positive compared with female patients [28, 29], and hormonal therapy is strongly recommended as treatment in these cases [24]. Triple-negative MBC is rare, representing only 3.6% of MBC, and has a significantly higher rate of recurrence and mortality compared with HR-positive breast cancer [30]. Further, increased incidences of MBC were also reported in obesity [31] and testicular cancer [32]. Other possible etiological factors in MBC development include drugs such as amphetamine use, head trauma (by increasing prolactin production), local chest trauma, previous radiation exposure, and smoking [25, 33, 34]. Classification and therapy of male breast cancer have largely been extrapolated from female breast cancer, because large clinical series of male breast cancer are lacking [35].

Recently several studies have suggested that the worldwide incidence of MBC is slowly rising [36–38]. Over the past 25 years, the incidence of MBC has increased by 26% [39]. Men are not well-educated regarding breast self-examination compared to women, which may be a reason for the high mortality rate among men with breast cancer. The late and often asymptomatic clinical presentation and rarity of the incidence of MBC preclude the use of early screening, and the disease usually presents at a more advanced stage compared with breast cancer in female patients, as it is not detected in time [40]. Furthermore, there is also no breast screening program for men, and the research on the genetic predisposition to breast cancer in men is currently limited [26]. Recently, several specialty hospitals in Saudi Arabia have started a national screening program, including one at our hospital, in the western region of KSA in Makkah city. In 4 years, 1,667 cases of female and male patients with breast complications were screened for this disease, among which 17 were men with various breast diseases, 2 of whom had invasive ductal carcinoma (IDC) of the left breast. Our aim is to present the analysis of the histological, ultrasound, and radiological findings and the prevalence of male breast cancer from the Makkah region of Saudi Arabia in this study.

## 2. Methods

### 2.1. Ethical Statement

This study was approved by the Institutional Review Board (IRB) for bioethics (IRB number HAPO-02–K-012-2020-12–508) and was performed in accordance with the principles of the Declaration of Helsinki. The patients were instructed with regard to the procedure, and a written informed consent was obtained from all patients or from their legal guardians before study initiation.

### 2.2. Patients' Selection, Demographics, and Data Analysis

From the total of 1,667 male and female patients screened by mammography cases during the period between July 2015 and April 2019, only 17 cases were with male breast diseases. The male registered cases were with breast complications, such as breast swelling and pain, and other breast abnormalities, who were referred to the Radiology Department of our Specialist Hospital in Makkah, Saudi Arabia, by the outpatient and inpatient departments. The diagnosis was made following the mammography and complementary ultrasound (US) and histopathological examination and immunology confirmation. Malignant tumors were classified according to the WHO classification of breast tumors series [41], and the Scarff-Bloom-Richardson (SBR) grading system was employed for tumor grading [42, 43]. Clinicopathological and demographic data were obtained for all patients and were analyzed for the present study. For statistical analysis, Statistical Package for Social Sciences (SPSS) version 22 was used. The quantitative variables, such as age, and the qualitative variables, such as mammography and US characteristics, immunomarkers, and histopathological diagnosis, were analyzed [44, 45].

### 2.3. Mammography and Ultrasound Examinations

Mammographic data were collected from a review of the radiology reports. Mammogram images had been acquired on a digital mammography machine (Mammomat Inspiration with PRIME Technology Siemens Healthcare, Germany). Two projections of both breasts were obtained using mediolateral oblique (MLO) and craniocaudal (CC) views. Specialized or magnification views were obtained wherever required. For each imaging modality, the findings were classified and interpreted according to the Breast Image Reporting and Data System (BI-RADS) assessment categories recommended by the American College of Radiology [46]. Following the BI-RADS classification, scores were allocated as follows: 0 for incomplete; 1 for negative, 2 for benign, 3 for probably benign, 4 for suspicious of malignancy, 5 for highly suggestive of malignancy, and 6 for known biopsy-proven malignancy [47]. The spectrum of malignant, benign, and likely benign screening mammograms was analyzed and assessed by the interpreting radiologist, along with the requirements for a corroborative US which were deemed necessary [48]. These included dense breasts, all cases of noncalcified lesions of solid density, and lesions corresponding to BI-RADS III.

### 2.4. Ultrasound-Guided Breast Biopsy Procedure

In cases of breast lesions, core needle biopsy (CNB) was preferably performed at our department utilizing a free-hand technique with a high-resolution US unit with 7.5 or 12 MHz linear array transducers (LOGIQ S8, GE Healthcare, Milwaukee, WI, USA). Each procedure was performed in an outpatient setting with aseptic technique, under local anesthesia, and with the patient in the supine position. A 14-gauge automated core biopsy needle with a spring-loaded biopsy gun (Disposable Core Biopsy Needle for BARD Magnum® System; Berlin Germany) and corresponding 13-gauge coaxial needle were used. Up to 4-5 samples were retrieved from the needle with the scalpel blade or sterile needle and placed in a vial with formaldehyde, and the sample was sent to the Pathology Lab for histopathological examination.

### 2.5. Postprocedure Care

Patients who had undergone the biopsy tolerated the procedure well, with no immediate complications, and were discharged from the department in stable condition. The patients were advised to apply compression to the biopsy site for 3-4 hours and avoid heavy lifting or strenuous activity for 72 hours. In case of bruising and tenderness of the breast, ice packing was advised over the next few days. Patients were advised to take acetaminophen (Tylenol) for discomfort or pain if needed and advised not to take aspirin or any other blood thinner for at least 72 hours.

### 2.6. Histopathological and Immunological Examinations

The excised tumor was fixed in 4% buffered formaldehyde, then processed for paraffin embedding. Four-micrometer-thick sections were prepared on clear ground glass slides and stained using Dako Reagent management system (DakoRMS) with hematoxylin and eosin (H and E) on a Dako CoverStainer (Agilent). For immunohistochemistry, sections were collected on Citoglas adhesion microscope slides. Antibodies against ER, PR, HER-2, Ki-67, and E-cadherin (Sigma-Aldrich, Ventana-Roche, and Leica Biosystems) were used for immunohistochemistry. Briefly, the tissue sections were deparaffinized with EZ Prep (Ventana, cat. no. 950-102) and immunohistochemistry was performed with the Ventana BenchMark XT automated Stainer (Ventana, Tucson, AZ). After inactivation of the endogenous peroxidase using a UV-inhibitor at 37°C, the primary antibody was added for 16 min at 37°C, followed by the application of HRP Universal Multimer for 8 min, and detected using the ultraView Universal DAB Detection Kit (cat. no. 760-500). The slides were counterstained with hematoxylin and bluing reagent before mounting with cover slips. Following staining, images were captured using Nikon Digital Microscope Camera-DS-Ri1, with image software NIS Elements v.4.0 [49].

## 3. Results

**Figure 1:**
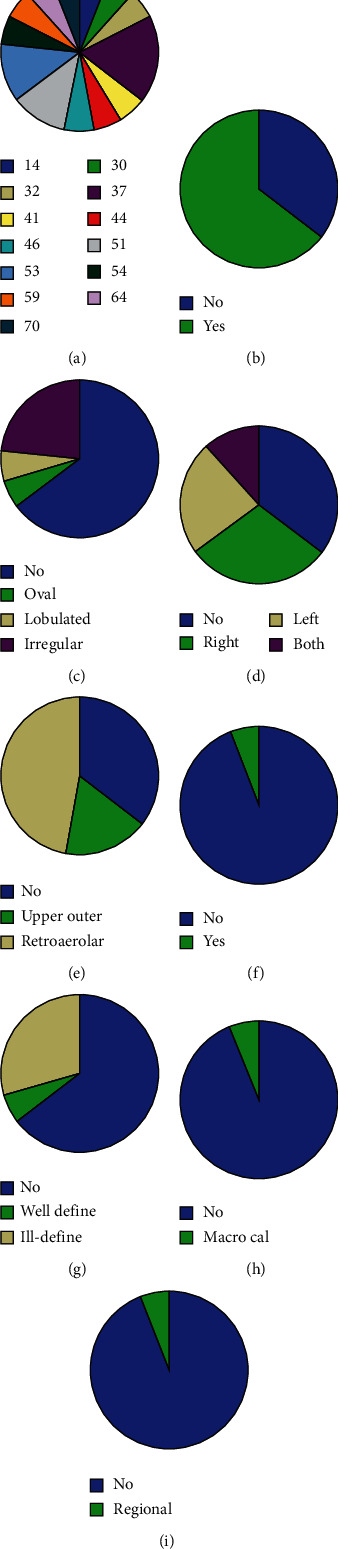
Characteristics of breast lesions found on mammography: (a) patient age (yrs), (b) presence of lesions, (c) shape of the lesions, (d) site of the lesion, (e) location of the lesion, (f) presence of skin thickening, (g) distribution of lesion borders, (h) distribution of breast calcification, and (i) presence of microcalcification.

A total of 17 registered cases were included for analysis; 16 of the patients underwent mammography, and 1 patient was screened with US only. The distribution of patients according to their age in this study population is shown in the pie diagram (Figure 1(a)). In the present study, 3 cases (17.6%) were aged 37 yrs; 2 cases each (11.8%) were aged 51 and 53 yrs; all other cases were one each in that age group, respectively. The mean age of the patients was 35 years in our study, and the minimum and maximum ages were 14 and 70 years, respectively. However, more patients (*n* = 11, 65%) were in the age group of 35-55 years and only three patients (18%) were in the age group of 56-70 years.

Of the 17 cases, 6 (35.3%) had no breast lesions and 11 (64.7%) had breast lesions (Figure 1(b)). One case had an oval-shaped lesion, a lobulated breast lesion was detected in 1 case, and irregularly shaped lesions were found in 4 cases (Figure 1(c)). A total of 5 (29.4%) cases had lesions on the right breast, 4 (23.5%) cases had lesions on the left breast, and 2 (11.8%) cases had lesions on both breasts (Figure 1(d)). The distribution of breast lesions according to their location is shown in Figure 1(e). Three (17.6%) cases had the lesion on the upper outer quadrant, and 8 cases (47.1%) had lesions with a retroareolar location. Skin thickening was observed in 1 (5.9%) case, whereas no skin thickening was observed in the remaining 16 (94.1%) cases (Figure 1(f)). Only 1 lesion (5.9%) had a well-defined border, and 5 lesions (29.4%) had ill-defined borders (Figure 1(g)). Calcification of breast was found in 1 patient (regional microcalcification; Figures 1(h) and 1(i)).

**Figure 2:**
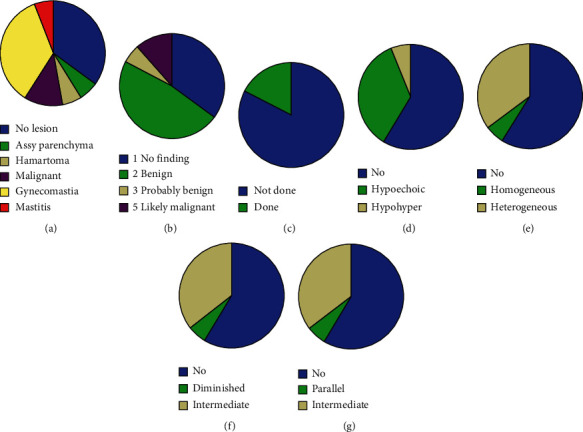
Characteristics of breast lesions found on mammography and ultrasound characteristics of breast lesions: (a) details of image diagnosis, (b) distribution of BI-RADS category, (c) biopsy status, (d) appearance of the lesion, (e) internal echoes, (f) acoustic transmission, and (g) axis of the lesion.

As regards the distribution of patterns and disease status of the lesions, there were 3 cases of asymmetry of the parenchyma, mastitis, and hamartoma (*n* = 1 each); 2 malignant lesions (11.8%); and 6 cases (35.3%) of gynecomastia (Figure 2(a)). A pie diagram showing the distribution of BI-RADS category from 1 to 5 is shown in Figure 2(b); 47.1% were benign BI-ADS (*n* = 8), 5.9% were probably benign BI-RADS (*n* = 1), and 11.8% were likely malignant BI-RADS 5 (*n* = 2). Biopsy was performed in 3/17 (17.6%) cases (Figure 2(c)). Ultrasound characteristics of breast lesions such as appearance of the lesion, internal echoes, acoustic transmission, and axis of the lesion are shown in pie diagrams in Figures 2(d)–2(g).

**Figure 3:**
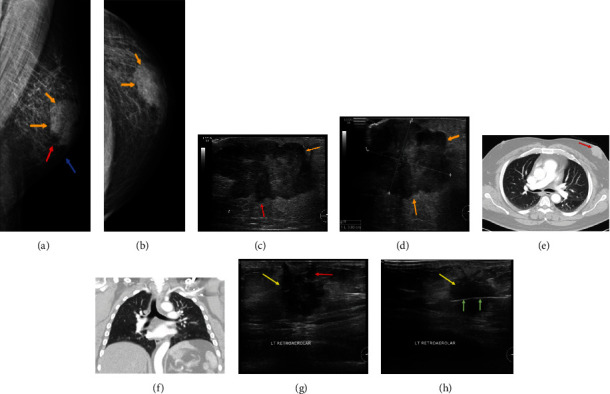
Left mammogram, ultrasound of the left breast, and chest CT of a male patient with malignant breast cancer. (a) MLO view and (b) CC view, showing a large dense mass (yellow arrow) in the retroareolar and upper location. The mass has irregular margins with adjacent coarse trabeculae (red arrow) and mild skin thickening (blue arrow). No microcalcification or skin retraction was observed. (c, d) Complementary ultrasound of the left breast revealed a huge mass (yellow arrow) with lobulated contours and heterogenous echoes. The lesion measured ~3.8 × 3.0 cm, was located at 1-2 o'clock position, and appeared to invade the adjacent parenchyma (red arrow). (e) CT images (lung window) of the first case showing a mass of the left breast (red arrow). (f) There was no evidence of metastasis to the lungs or mediastinum. (g, h) Show the ultrasound-guided left breast biopsy of the second malignant case. (g) A huge hypoechoic mass (yellow arrow) with lobulated contours. This is located at 1 to 2 o'clock position and appears to be invading the adjacent parenchyma (red arrow). (h) Shows the tumor mass (yellow arrow) and biopsy needle (green arrow).

In the present analysis, 2 cases with malignant lesions were identified. The first case was in a 54-year-old male patient, and the second case was in a 64-year-old male patient. The first patient was a known case of diabetes mellitus on medication, who presented to the “Emergency Room” complaining of a left breast mass for 4 years and a history of trauma 8 years prior due to a traffic accident. Bilateral mammography was performed, and CC and MLO views of both breasts were obtained (Figures 3(a) and 3(b)).

The left breast displayed a huge dense mass lesion in the upper retroareolar area, with coarse trabecular pattern and mild thickening of the periareolar skin. There were no clumps of microcalcification or retraction of the skin, but a lymph node was noticed in the axilla. The mammography revealed a highly suspicious left breast mass (BI-RADS 5). The right breast exhibited no dominant mass, suspicious clumps of microcalcifications, skin thickening, or retraction. Retromammary and axillary locations were found to be normal. Complementary ultrasound of the left breast revealed a huge mass with a lobulated contour and heterogeneous parenchyma (Figures 3(c) and 3(d)).

The mass was located at the 1-2 o'clock position and measured >3.8 × 3.0 cm. In addition, enlarged axillary lymph nodes were identified, with preserved fatty hilum, with one of the lymph nodes exhibiting cortical thickness of ~0.2 cm. Complementary CT examination of the chest, abdomen, and pelvis was conducted to rule out distant metastasis. The CT images revealed a large lobulated mass in the left breast, with mild thickening of the overlying skin (Figure 3(e)). There was no mediastinal, hilar, or internal mammary lymphadenopathy (Figure 3(f)). The findings of the right breast were unremarkable, and no metastatic lesions were observed in other parts of the body. A Tru-Cut biopsy using 16-gauge inner cutting needle was done under US guidance from left breast lesion at the retroareolar position. Three tissue samples were collected and sent for histopathological analysis. Two months after mastectomy, the patient was subjected to a lumbosacral spine MRI for back pain, which revealed spondylosis type of changes and bulging discs, but no evidence of bone metastasis.

**Figure 4:**
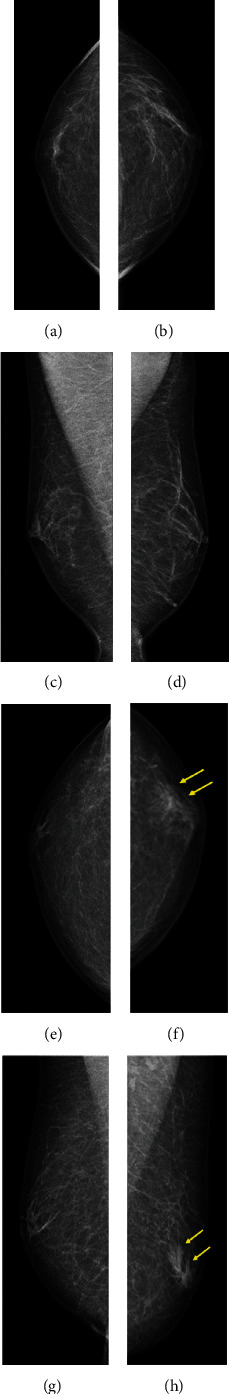
Normal bilateral mammograms and bilateral mammograms showing left breast gynecomastia. (a) RCC, (b) LCC, (c) RMLO, and (d) LMLO views, respectively. There are no masses or parenchymal distortion and suspicious calcifications or skin thickening. (e) RCC, (f) LCC, (g) RMLO, and (h) LMLO views, respectively. Left breast mammogram shows dendritic subareolar density with posterior linear projections radiating into the surrounding tissue toward the upper-outer quadrant (yellow arrows), suggesting chronic dendritic gynecomastia (f, h). Right breast mammograms (e, g) appear normal.

The second patient presented with a lump in the left breast, and US-guided breast examination (Figures 3(g) and 3(h)) revealed a huge dense mass lesion in the upper retroareolar area, with a coarse trabecular pattern, mild thickening of the periareolar skin, and an enlarged lymph node identified in the axilla. Retromammary and axillary locations were normal, and no clusters of microcalcification or retraction of the skin were observed. A representative mammogram of a normal breast (BI-RADS 1) from a 31-year-old male patient with history of left breast swelling is shown in Figure 4. The bilateral mammogram (a) and (b) (CC) and (c) and (d) (MLO) views revealed no dominant mass lesion, suspicious clumps of microcalcifications, parenchymal distortion, skin thickening, or retraction. A representative case of gynecomastia in a 69-year-old male patient is shown in Figures 4(e)–4(h). Mammography of the left breast revealed a small retroareolar soft-tissue density radiating into the deeper adipose tissue, with no detectable mass or pathological calcification. Two small left axillary lymph nodes with central hypodensity suggestive of benign nature were present (f and h). No abnormal density or masses were observed in the right breast ((e and h). In the left breast, a small stranded density suggestive of dendritic fibrous gynecomastia was observed (BI-RADS 2 category).

**Figure 5:**
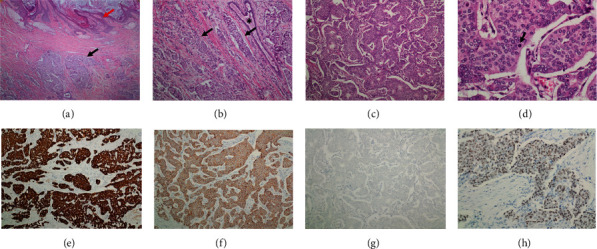
Representative hematoxylin and eosin (H and E) staining and immunohistochemistry staining of male breast cancer. (a) Invasive carcinoma (black arrow) can be seen immediately below the skin of the nipple (red arrow), with no evidence of skin invasion; magnification, ×40. (b) Infiltrating nests of tumor cells (arrow) surrounded by desmoplastic stroma. The asterisk indicates the entrapped normal duct; magnification, ×100. (c) Invasive ductal carcinoma composed of malignant cells arranged in sheets and nests; magnification, ×100. (d) High-power view showing malignant cells with intermediate-grade nuclei that exhibit mild to moderate pleomorphism, open vesicular nuclei, and punctate nucleoli. Frequent mitotic figures were also seen (arrow); magnification, ×400. (e) Staining for pancytokeratin exhibited diffuse positivity; magnification, ×100. (f) E-cadherin expression exhibited a membranous staining pattern on H and E staining; magnification, ×100. (g) P63 was completely negative, which indicates loss of the myoepithelial layer and confirms the invasive nature of the tumor; magnification, ×100. (h) Estrogen receptor staining exhibited intermediate to strong nuclear positivity in >90% of tumor cells; magnification, ×200.

Histological examination of the first case identified on microscopic examination as malignant revealed an invasive neoplastic mass composed of malignant cells arranged in sheets, nests, and strands, with moderate nuclear atypia. Hematoxylin and eosin (H and E) and immunohistochemical staining revealed IDC of no special type (NST), grade II. Representative sections are shown in Figures 5. In Figure 5, panel (a) revealed invasive carcinoma just below the skin of nipple, with no evidence of skin invasion. Infiltrating nests of tumor cells were surrounded by desmoplastic stroma, with entrapping of the normal duct, and the malignant cells formed sheet- and nest-like structures (b and c). High-power view examination revealed malignant cells with intermediate-grade nuclei that exhibited mild to moderate pleomorphism, open vesicular nuclei, and punctate nucleoli with frequent mitotic figures (d).

**Figure 6:**
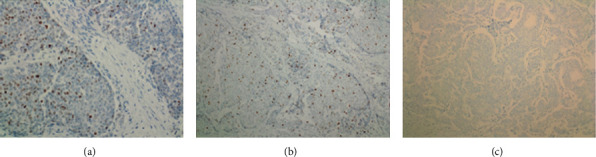
Immunohistochemistry staining of male breast cancer. (a) Progesterone receptor staining was positive in 30% of tumor cells (lower percentage compared to estrogen receptor); magnification, ×200. (b) The Ki-67 proliferative index was 20-30%; magnification, ×100. (c) HER-2 was completely negative; magnification, ×100.

Immunohistochemical staining for pancytokeratin was strongly positive (magnification ×100; Figure 5(e)); E-cadherin expression was positive, with a membranous staining pattern (Figure 5(f)); P63 was completely negative, which indicated loss of the myoepithelial layer and confirmed the invasive nature of the tumor (Figure 5(g)); estrogen receptor (ER) exhibited intermediate to strong nuclear positivity in >90% of tumor cells (Figure 5(h)). As shown in Figure 6, progesterone receptor (PR) staining was positive in 30% of tumor cells, which was a lower percentage of tumor cells compared with ER (a); Ki-67 proliferative index was positive in 20-30% (magnification ×100; (b)). As shown in Figure 6(c), HER-2 was completely negative.

**Figure 7:**
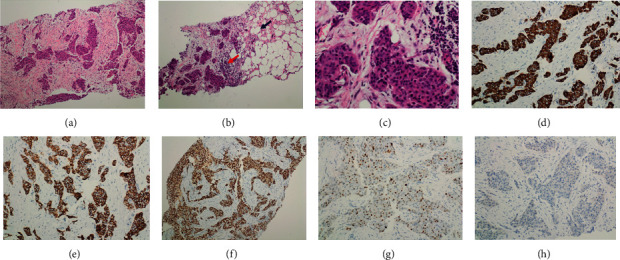
Histopathology and immunology of the needle core biopsy of the malignant tumor. (a) Biopsy tissue showed invasive ductal carcinoma forming solid sheets and nests of tumor cells surrounded by desmoplastic stroma, H and E, ×100. (b) Nests of tumor cells infiltrating adjacent fatty tissue (black arrow). Note the stromal lymphocytic infiltrate (red arrow), H and E, ×100. (c) High-power view revealed tumor cells with intermediate-grade nuclei, H and E, ×400. (d) Pancytokeratin is strongly positive in tumor cells. (e) E-cadherin stain showed strong diffuse membranous positivity, ×200. (f) ER showed strong nuclear positivity in more than 90% of tumor cells, ×200. (g) PR showed positive nuclear staining in around 30% of tumor cells, ×200. (h) HER-2 is completely negative, ×200.

As shown in Figure 7, a needle core biopsy performed 1.5 months prior to the mastectomy also confirmed the diagnosis of IDC. Formation of solid sheets and nests of tumor cells surrounded by desmoplastic stroma is shown in H and E staining (a); furthermore, nests of tumor cells infiltrating adjacent fatty tissue were identified (b). There was also a stromal lymphocytic infiltrate (b), and high-power view examination revealed tumor cells with intermediate-grade nuclei (c). Pancytokeratin was strongly positive in tumor cells (Figure 7(d). The E-cadherin staining exhibited strong diffuse membranous positivity (Figure 7(e)), ER exhibited strong nuclear positivity (Figure 7(f)), PR exhibited positive nuclear staining (Figure 7(g)), and HER-2 was completely negative (Figure 7(h)). The sentinel lymph node was examined and found to be negative for metastasis. Histopathological examination of the second case (IDC grade III) revealed infiltration of the breast parenchyma by malignant epithelial cells arranged singly in cords, tubules, and sheets. The malignant cells exhibited marked pleomorphism, increased nuclear-cytoplasmic ratio, and increased mitosis, whereas the stroma exhibited prominent hyalinization. This tumor was positive for ER, PR, E-cadherin, p63, and Ki-67 (40%) and negative for HER-2.

## 4. Discussion

Breast cancer can also occur in men, and although it is not as common as in women, there is currently a momentum in reporting MBC cases from several countries [10, 11, 33–37]. According to the National Cancer Institute, 2,360 men were diagnosed with breast cancer and 430 men succumbed to this disease in 2014. Accurate data on the prevalence and mortality of breast cancer in men are not available from many Middle Eastern countries, including KSA [50, 51]. According to publications by the American Cancer Society (ACS), the 5-year survival rate of men with breast cancer is 97%; if the cancer is located only in the breast, about 47% of cases are diagnosed at this localized stage. However, the 5-year overall survival rate for men with breast cancer is 84% [52]. The positive ER/PR status is associated with more favorable 5-year survival rates in patients with MBC [53]. Nodal and distant metastases (visceral and skeletal) were reported to be associated with a worse prognosis of MBC [54]. In the cases studied herein, histopathological examination of the sentinel lymph node revealed no metastasis, which was also confirmed by CT results.

The Surveillance, Epidemiology, and End Result (SEER) Program reported that the incidence of breast cancer was highest at ages 52-71; this is in agreement with our data [38]. The age of MBC diagnosis in our cases (54 and 64 years) was in accordance with that reported from many ethnic regions of the world. In a retrospective analysis of 27 North Indian MBC patients, the average age found was 62.6 (range 46-77) years; also, another study from Eastern India reported that the mean age of male patients was 56 years, range being 30–78 years (*n* = 42). The largest number of malignant cases was found in the age group of 41–50 years [55, 56]. Another study from Turkey reported that the median age at presentation was 62 years (range, 35-90 years) [57]. The mean age of the male breast cancer incidence was 63.3 years in Japanese males; however, at diagnosis, the mean age of Japanese MBC patients was 70 years [58, 59], whereas in US MBC patients, the mean age at diagnosis was 67 years [60]. In the Southern China region, the age at diagnosis in male patients was 64.5 years, whereas in the northern China region, it is 62 years (range, 24-84 years) [61, 62].

In the present analysis, out of a total of 17 male cases, only 2 cases had MBC of the IDC type. From a histological point of view, all microscopic types of breast cancer may develop in men. According to WHO classification of invasive MBC [41, 63], the most common type is IDC-NST, intermediate- to high-grade. Papillary carcinoma, although very rare, is relatively more common in men compared with women [24]. Lobular carcinoma is extremely rare due to absence of terminal duct lobular unit (TDLU) in the normal male breast [64]. Other types, such as mucinous, secretory, tubular, and metaplastic carcinomas, are exceedingly rare.

The immunohistochemical profile of IDC-NST reveals diffuse positivity for cytokeratin (CK). E-cadherin exhibits strong circumferential membranous staining, except in lobular carcinoma, in which there is loss of staining due to mutation of the CDH1 gene. CK is expressed in >90% of breast cancer, whereas CK20 is mainly expressed in gastrointestinal tumors [65]. According to the IHC-based categorization of breast cancer subtypes [66], the majority of MBCs are luminal A subtype (ER^+^ and/or PR^+^, HER-2^−^, and Ki-67 low), followed by luminal B subtype (ER^+^ and/or PR^+^, HER-2^+^, and/or Ki-67 high). For instance, MBC positive for HER-2 is rare which is around 1.7%, and triple-negative and basal-like cancers are also rare [67]. Here, we reported a case of luminal A type in this study. In the second case, histopathological examination of the tumor revealed infiltration of the breast parenchyma by malignant epithelial cells arranged singly in cords, tubules, and sheets. The malignant cells exhibited marked pleomorphism, increased nuclear-cytoplasmic ratio, and increased mitosis, whereas the stroma exhibited prominent hyalinization. On immunohistochemical staining, the tumor was positive for ER, PR, HER-2 (+3), E-cadherin, p63, and Ki-67 (40%). The final diagnosis was IDC grade-III, luminal B type in this patient.

The symptoms of MBC are similar to those of breast cancer in women, a palpable mass and enlarged lymph nodes or skin changes (ulceration and secretion). The MBC symptoms are typically a painless mass, occasionally associated with nipple retraction or ulceration and bloody nipple discharge [68]. In accordance with previously published data, positive correlation of high Ki-67 index with high-grade tumors and larger tumor size was noticed in our study in one patient [69]. MBC is frequently established in more advanced stages [70]. MBC most commonly develops in the central retroareolar/nipple area which has the greatest lymphatic drainage in the breast and rarely has chest wall involvement or skin invasion [33, 57, 71, 72]. In terms of tumor site, 51.2% left breast and 48.8% right breast were reported [57]; however, some studies reported left breast is predominant [71]. Our two malignant patients are also left breast cases and without nipple retraction. We have found only 1 patient who had a well-defined mass, and 5 patients had ill-defined mass out of total 17 screened cases that is closely similar to the data reported by Doyle et al., where they found that out of 13 patients who underwent mammography, 6 of them had an ill-defined breast mass and 2 had a well-defined breast mass visualized [73]. The time interval between the onset of complaints and admittance to hospital ranged from three months to two years [74]. The median duration of the evolution of the MBC symptoms and signs was 9 months [75, 76]. In one of our cases, the patient noticed nipple discharge that became bloody over time after a traffic accident, for which he sought treatment 1 year prior to mammography. Also, a Spanish study reported that the average delay between symptom onset and diagnosis was >10 months [77]. Our study attempted to present the radiological and clinicopathological characteristics of MBC patients in the western region of KSA. According to imaging features of the 20 patients who had an ultrasound, 16 (80%) lesions were presented as hypoechoic solid masses with irregular margins [74]; in our cases, also, majority of cases have ill-defined margins and hypoechoic solid mass, respectively.

Literature pertaining to MBC is overall sparse, particularly in Saudi Arabia and other Middle Eastern countries. A single case report of occult triple-negative MBC originated from Bahrain [75], and a study describing 5 MBC cases of papillary-type DCIS and a case report of triple-positive IDC stage IIIB were reported from KSA [76, 78]. A retrospective analysis over 8 years including a total of 1,005 breast cancer cases in Saudi patients in the Madinah region of KSA found that 23 were MBC cases, 4 of which (17.4%) were IDCs [79]; also, another nineteen years of retrospective analysis of 15 MBC cases is recently published; in that, also, the most predominant type of MBC was IDC, which affected 14 patients out of 15 [80]. In the retrospective analysis of 37 cases of MBC from Egypt, also, 94.5% of the tumors were IDCs in their study [71].

## 5. Conclusions

The main limitations of this study are its retrospective design and the limited number of cases because of the rarity of this disease. Our results are in accordance with published data in terms of age at diagnosis, histological cancer type, tumor grade, hormone receptor expression, and duration of symptoms till the time of final diagnosis. However, we do not have data on family history, long-term survival, and expression of AR also. Our sample number of MBC cases is very low to compare with other studies to do a thorough analysis. The pathological and immunocytochemistry examination revealed IDC-NST, grade II and grade III, which is in agreement with the literature suggesting that IDC is the most common type of MBC. Genetic analysis to determine the BRCA1/2 mutation status is lacking in our study; however, we reported the HER-2 status. In the present study, the average age of the patients was 35 yrs, whereas only 18% were in the age group of 56-70 yrs, which is contradictory to the median age of 57.7 and 62 yrs previously reported in retrospective studies; this discrepancy is because only two cases are malignant.

Multicentric large prospective investigations are lacking in MBC area, and there are not many reports of comprehensive analysis of MBC from Saudi Arabian population and in general from many Gulf countries. This present study may help with increasing awareness of MBC in Saudi Arabia and encourage men to learn to perform self-breast exam and undergo routine screening by mammography if suspicion arises. Establishing a Middle Eastern region's prospective MBC registry that will collect tissue specimens and diagnostic and treatment information in order to answer critical clinical questions is essential in this time. These future research efforts will facilitate the development of interventions that improve the prognosis of individuals in this unique and understudied population.

## Data Availability

The datasets used and/or analyzed during the current study are available from the corresponding author on reasonable request.

## Abbreviations

ACS: American Cancer Society
AR: Androgen receptor
BI-RADS: Breast imaging reporting and data system
CC: Craniocaudal
CT: Computerized tomography
DAB: 3,3′-Diaminobenzidine
DCIS: Ductal carcinoma *in situ*
ER: Estrogen receptor
PR: Estrogen receptor
H and E: Hematoxylin and eosin
HER-2: Human epidermal growth factor receptor 2
HR: Hormone receptor
HRP: Horse radish peroxidase
IDC: Intraductal carcinoma
IHC: Immunohistochemistry
KAMC: King Abdullah Medical City
KSA: Kingdom of Saudi Arabia
MBC: Male breast cancer
MLO: Mediolateral oblique
NST: No special type
TDLU: Terminal duct lobular unit.
